# Supplementary material for: A high-resolution mRNA expression time course of embryonic development in zebrafish
Source: eLife. 2017 Nov 16;6:e30860. doi: 10.7554/eLife.30860 (PMC5690287; doi:10.7554/eLife.30860)
Supplement: Supplementary file 6. [file elife-30860-supp6.zip › biolayout-clusters-files/Cluster157-genes.html]

Cluster157


# Cluster157: Genes

| Ensembl ID | Gene Name | Chr | Start | End | Biotype |
| --- | --- | --- | --- | --- | --- |
| ENSDARG00000027609 | HPN | 16 | 45282339 | 45321883 | protein\_coding |
| ENSDARG00000057714 | cmah | 4 | 2379441 | 2400951 | protein\_coding |
| ENSDARG00000061365 | hepacam2 | 19 | 40763348 | 40787308 | protein\_coding |
| ENSDARG00000060854 | kctd3 | 17 | 29328643 | 29353351 | protein\_coding |
| ENSDARG00000035682 | ltbp3 | 5 | 36917524 | 36985323 | protein\_coding |
| ENSDARG00000015278 | plxnc1 | 4 | 18293670 | 18328628 | protein\_coding |
| ENSDARG00000034677 | scel | 9 | 17298450 | 17326946 | protein\_coding |
| ENSDARG00000094422 | si:dkey-12l12.1 | 24 | 11194366 | 11206504 | protein\_coding |
| ENSDARG00000001354 | slc6a13l | 6 | 41146667 | 41163481 | protein\_coding |
